# President’s Address

**Published:** 1896-11

**Authors:** B. Fincké

**Affiliations:** Brooklyn, N. Y.


### PRESIDENT'S ADDRESS.

B. Fincké, M. D., Brooklyn, N. Y.

(Concluded from page 433.)

The physico-chemical character of the reigning (not regular) old medical school has not changed at all. Diphtheria is caused by a poison produced by a parasite called the Diphtheria-bacillus. The more poisonous the bacillus, the more poisonous must be the antidote which they conceive to be the remedy. The stronger poison must subdue the weaker, and so the antitoxine taken to be the stronger poison must be increased to double and more of its strength and inoculated in greater quantity. Whether the human living organism can stand it does not seem to bother them in the least. If it cannot stand it it is the fault of the organism, not of the intelligent, rational therapeutist, who stands excused from any intentional or unintentional murder before the law of the land. This is the unfortunate domination which chemistry of late has gained upon the department of internal medicine as art and science of healing. Without taking heed of organic life in the body from disease, they treat man like a retort, in which they mix their poisonous substances taken from disease and death. They assume duties for which their physico-chemical education does not fit them. What are we coming to? Must the homœopathic physicians submit to the ignominious position assigned to them in these modern times by the allopathic authorities to apply remedies manufactured by them which are mere caricatures of medicine as bad as the mediæval products of the then recommended dirt-pharmacies (Dreckapotheken) in the diseases pronounced infectious and contagious, and depending upon pathological speculations which do not bear close investigation? Must the physicians carry out what chemists, going out of their sphere from unproven premises and false principles, prescribe in diseases, because by the microscope they have found a miniature world of scavengers and parasites in sick bodies which, if not counteracted by proper neutralization, is said to involve it in unavoidable destruction? There are more things to be considered than to place the morbid product under the microscope and make it the basis of a prescription. This seems to be curing made easy; but it will be turning out no curing at all, but spreading and aggravating disease, and killing.

What perversion of the mind has taken possession of the medical men of the old school at the end of this glorious century? What warping of judgment constantly is going on as if lunatics were at the head of the faculty, and not learned men having passed through long years of study and examinations and re-examinations. If a person in private life would go to work and inject into the body of a healthy child a substance recommended by high authority to prevent a certain disease, and two minutes afterward that child dies, should not the law step in and accuse the person of the crime of poisoning that child and causing its death? Should it not be declared manslaughter? Does it change the matter any if that person is a physician entitled by the high authority of a sanitary body to do such a thing? Nay, he should be held doubly responsible, because the State has given him license to cure the people or mitigate their sufferings, but not to kill them. What a monstrous perversion of intellect! Must this maiming and killing of the innocents continue in the coming centuries? It is a well-known fact that the fanatics of vaccination never admit that from its operation spring copious diseases, and many cases of death caused by it are stated upon the best evidence, but they will never deviate an iota from the advice of Jenner, to conceal the ill-effects of vaccination from the public. What a fiendish advice! What a state of morals among the fraternity of learned men whom the public revere as their saviors on this earth, placing their life and health confidingly in their hands! It is incredible but true; the crude empirical procedure of the allopathic school has given rise to a degree of mendacity which makes all their statistics doubtful, and statements in medical matters must be received with utter scepticism till proved to be true. What a contrast, on the other hand, is presented in looking at their treatment of Homœopathy! Whilst they are not afraid of administering to the sick poisonous doses of crude medicines and morbid substances, inoculating them into the body, they grudge to the homœopaths the use of their potencies, which derisively they call nothings, and in Germany force them to prescribe their potencies in the licensed shops, compelling them to undergo an examination by which only they can acquire the right of self-dispensation, and to submit to the arrogance of a high official, who makes it a favor to be bestowed. Nay, it is forbidden in some countries to give simple powders of sugar of milk and globules on the part of the physician. May the good God protect us from such unmitigated despotism on the part of a profession which calls itself the regular school of medicine, and is so irregular in its practices! We have seen compulsory vaccination taking hold of late years in our blessed country where it never was before, and all efforts have been in vain so far to defeat this indignity and outrage upon a great nation, because the people are kept in ignorance and fear of that dreadful disease, which on the whole does not undermine the public health as much as vaccination does. May the physicians of our country look out that the precious right acquired by competent study, and the privilege given by the Commonwealth to practice according to our best ability, be not curtailed and gradually altogether abolished.

There is no scientific foundation to the old school, as has been laid down for Homœopathy a hundred years ago by Hahnemann. It only proceeds upon empirical data and traditions and methods purloined from the chemical laboratories which give them not a particle of right to domineer over our new school. If the surgical branch has made enormous progress in the way of operating, it cannot change the homœopathic conception of medicine. Rather surgery can learn from it how many operations can be prevented or assisted favorably by homœopathic treatment. If surgery has been progressive, Homœopathy has not been stationary either; though its law is eternal, it certainly goes forward in perfecting the materia medica pura and posology. Through the introduction of the high potencies for the last fifty years, the range of healing has been remarkably widened, and promises still further favorable results in curing diseases even thus far deemed incurable, and in mitigating the amenable ones. We have often heard that the elder homœopaths have been more successful than those of our time in this respect. With all due honor to the pioneers living in a time when the enmity of allopathy was fiercer than now; when the medicines of homœopathic physicians were confiscated and buried in a churchyard; when homœopathic physicians were prosecuted in court by great professors to cover their own misdeeds; when they accused Hahnemann of murder because he would not bleed in inflammation of the lungs—and, what might we say now, looking upon a case of injection of anti-toxine killing a healthy child in two minutes—we do not think so. Witness the cases reported in our journals for the last thirty years, and in the many volumes of the International Hahnemannian Association, and you will admit that we do yet march abreast with our predecessors in the direction of equal if not greater efficiency in healing. It is pitiable that we have no more cases reported by our greatest men, because the recorded facts of clinical experience are the very rock upon which the science of homœopathics is being built up. We do not think that cases recorded lead off from earnest study of the materia medica; quite the contrary, they give it more interest and stimulate the healer to do likewise. Clinical cases are, therefore, mostly to be desired as stepping-stones to greater perfection. But of course, reports of cases treated in the crude allopathic manner, and of cases which present no interest but the skill in operating in surgery and gynæcology without any ostensible relation to homœopathic treatment, would be here in the wrong place. They can be vented in the circles which will receive them with applause, but are foreign to us, to say the least.

There is, however, a point to be considered which is apt to throw a false light upon our work, and that is what generally is admired as a broad, liberal view, embracing everything without a principle, but is only a reprehensible latitudinarianism, as Goethe says:

Getretner Quark wird breit, nicht stark.

The International Hahnemannian Association wisely carries in its motto the "*minimum*," mindful of Hahnemann's inculcation of the least possible dose, which we encounter in his earliest writings, as above mentioned, as far back as in 1801, where he says: "Will they at last understand how small, how infinitely small, the doses of the remedies need be in the sick state in order to affect the body powerfully?" and in 1805: "This dynamical action of the medicines is as the vitality itself by which it is reflected upon the organism, almost purely spiritual, most strikingly that of the positively (curatively) employed remedies, with the peculiarity that the too strong dose can indeed do harm and produce considerable disorder in the body; but a small dose, a most possibly smallest one, cannot be unhelpful if the remedy is otherwise well indicated."

But if the doses at that time were already comparatively small in relation to those of the old school, Hahnemann did not continue to rest on this standpoint, as the friends of low potencies contend, but went steadily up higher in the scale of potentiation till, at the time of the first appearance of higher potencies than he had ever used, he hailed the new discovery with the ardor of a young man even in his old age (seventy-seven) as the fulfillment of his earlier predictions. Now, the great majority of the homœopathic profession confessedly adheres to the false representations that Hahnemann had always advocated and used the so-called low potencies and dilutions, or triturations, which they use even now in the decimal scale. There are some among them who let well enough alone, and use the lowest and the higher potencies as they see fit, and thus a rule has originated: the whole scale of potencies from the lowest to the highest must be at the disposition of the homœopathician. This, indeed, sounds quite liberal and broad, but it is not Hahnemannian; for Hahnemann nowhere said such a thing. Bœnninghausen stigmatized it "as an empty phrase designed to deceive the ignorant, as long as sure rules, resting upon irrefutable experience, are wanting, according to which this or that potency deserves to be preferred and is to be selected. Of such guiding rules we hitherto have not been able to find anything but the above-mentioned one, the falseness of which is evident and, at the same time, moves within very limited spaces." Quite in opposition to those false assertions, after the experience of a long life—and what an experience his was—Hahnemann laid down the rule that provided the correct selection of the remedy according to symptoms-simility, the dose can never be small enough still to overcome the disease. This is what our "*minimum*" means, and if we will follow the master of our art in other things, we must follow him also in posology.

In thus following our own course we do by no means reject those who still dwell upon the lower rungs of the ladder, and invite them to come up to a higher perception of homœopathics, such as is the true spirit of Hahnemannian Homœopathy, by our institution of junior membership. By it the applicant is not required to indorse the Declaration of Principles at once, but has the privilege of the floor for the discussion of medical topics and of presenting such papers as are indorsed by the Board of Censors, and is entitled to a copy of the transactions. If, then, after three years of attendance he wants to stay with the International Hahnemannian Association, he is welcome to active membership, subject to the necessary conditions. If he doesn't want to go on, why there is no necessity for it, and he simply drops off, a thing, however, which thus far has never happened. It is certainly true that the low potentialists are much more liable to fall back upon the doubtful resources of allopathy in critical cases, and use means condemned in no unmistakable language by Hahnemann.

They are also more inclined, as a general rule, to make pathological reasoning their guide, which, as pathology always tends to generalizing and adapting cases to the patterns of diseases arrived at by it, is apt to lead the homœopathic physician to neglect the necessary individualization of each case according to its symptoms. This necessity of healing, the proper taking of the case in all its details, and consideration of the value of the different symptoms in regard to similar symptoms of the pathopoëtic medicine, will always be the essential duty of the homœopathician. The difficulty of coming up to this requirement deters him who looks at disease as an entity for which he must find a counter-picture in the pathology gotten up by the old school containing patterns of every disease, which he has only to adapt to his case in order to work the miracle of a cure in which he is sure to fail. The salient point in the pathological description is not always the salient point in a pathogenetic picture for which we are bound to find the appropriate remedies. The pathological patterns are generalizations drawn from a multitude of cases during life under allopathic treatment with crude substances, large doses and injection of morbid substances, and derived from post-mortem examinations of the diseased parts of the body, and consequently they cannot govern our treatment of the individual case before us. Pathology takes very little heed of the subjective symptoms which play the most conspicuous part in our purpose of healing, for they are the signs of life, the manifestation of the life-force which through them calls to us for help. On the other hand, our remedies have been proved upon the live, healthy body, and the subjective symptoms, as a general rule, are of greater importance than the objective ones which the physician can, and of course must also observe; nay, the subjective symptoms frequently acquire an objectivity which leaves the observation of objective symptoms far behind.

One of our best men contends that Homœopathy is a pathological science because it deals with the cure of diseases. Hahnemann has long ago refuted such a narrow view when he wrote in the first paragraph of his *Organon*, even in the first edition in 1810: "The physician has no higher aim than to make sick men well." The difference, little as it appears at the first glance, is a great one between treating a disease in an individual and treating the sick individual himself. The disease is treated as an individual according to pathological knowledge, and the living individual is eclipsed. If Hahnemann taught to heal by a ὅμοιον πάθος *homoion pathos* (similar suffering), he did not mean by the pathological codex laid down by the old school, but by the knowledge of healthy individuals made sick by medicines in order to learn its pathopoëtic symptoms. All the knowledge conveyed by our materia medica pura is composed of the collection of these pathopoëtic symptoms observed in many individuals, which, when arranged in a convenient order, reveal the peculiar character of making people sick in the direction of the force which the medicine is able to exert. To this collection is added what medical writers on the positive actions of the medicaments have recorded.

This is a pathology quite different from what is called pathology in the old school, and is a branch of medical science worth studying more than the accumulated theories of allopathic pathology, since it tends to better knowledge in healing the sick who apply to us for help but not for pathological speculation. Thereby is not meant that the pathological study is not necessary and pathological knowledge need not be cultivated and increased. Certainly not. The study of pathology is as necessary as the study of physiology to a certain extent; but it should not take the lead in our endeavor to heal the sick. The efforts to find the pathopoëtic actions of medicines by poisoning animals and cutting them up alive belongs to the savage science, which, unfortunately, too many embrace under the apprehension of increasing the realm of true science. It leads to a degeneration of the medical profession which delights in cutting and slashing and numerous operations, but in the ordinary mind gradually deadens that sensation of human fellowship which should never be forgotten, even when treading the highest pinnacle of science. How many sick people are sacrificed to this moloch of a savage science which loves to maim and kill nobody knows better than the homœopathicians, to whom they afterward come for help when they escape, or from whom they depart under the erroneous impression that the homœopathic treatment is insufficient, in order to be taught a dreadful lesson on the operating table of the surgeon and gynæcologist. You are well aware that there are certain limits where, without doubt, surgical treatment is required; but the knife should always be the *ultima ratio* of the physician as the sword is that of the kings. These limits should never be overstepped. It has been the aim of Homœopathy from its beginning to remove those limits as far as possible, and we are happy to say that there are many surgeons and gynæcologists in our ranks who, though up to date in the proficiency of their specialty, are in full accord with this effort. There must always be a critical line which requires a keen judgment to demarcate. We have been told that—*e. g.*, in appendicitis—the surgical treatment should step in when there is yet hope for a successful termination. The same is said of croup, incarcerated hernia, as also in poisoning, in bites of venomous animals. This sounds quite reasonable. But where is that debatable line? Every medical man must decide for himself about it and act accordingly. But it should not be left out of sight that Homœopathy, if properly understood, with assistance of its rich materia medica pura and in the possession of the best homœopathic potencies of all grades, has an enormous advantage over the operator who has only a limited or no homœopathic knowledge, though his surgical acquirements be without limitation. We need the surgeons, we need their exact anatomical knowledge, their keen judgment, their skillful hands, their steady purpose, their kind management of serious cases requiring their aid; we want to attach them more and more to the art and science of homœopathics, in order to reach that high goal expressed in the old adage: *Salus asgroti suprema lex.* (The well-being of the sick is the highest law.)

Every one has his own gift; one is a good prescriber, another a good prover, another a keen searcher in philosophy, another excels in surgery, another in gynæcology, another cares for hygiene, another is proficient in preparing medicines, and so on. If every one does his best in following out his predilections and natural gifts always within the principles of homœopathics, which we one and all profess as the principles of healing, our noble cause must progress victoriously and gradually spread its insensible action like a high potency working throughout the human world, and finally also bring those to their senses who now, with the aid of public ignorance, political influence and power exerted in the wrong direction, try to extinguish the light which our own Hahnemann has kindled just one hundred years ago. They will never, *never* succeed!

Looking at the enormous progress in the departments of physics, chemics, and surgery, a striking contrast is presented when observing the shortcomings of internal medicine in the physico-chemical school which are deplored by the physicians belonging to it themselves. The shocking mortality in diphtheria, in contrast with the small percentage of death under homœopathic treatment, have forced the allopaths to adopt a mode of treatment neither isopathic nor homœopathic, as shown above, but true to its old standard, simply allopathic. If thereby the rate of mortality is lowered it must, in the interest of those who are to be saved by it, which is still doubtful, be hailed as a progress, though very insignificant in proportion to homœopathic success.

The late discovery of Rœntgen of the penetration of the negative electric rays through solid bodies in the dark tube exhausted of air, connected with pathopoëtic effects upon the operator, shows how the attenuation of air under the air-pump gives the electricity conveyed to it an opportunity to exert an energy which escaped the observation in broad daylight and in the open air. Yet these cathodic rays no doubt act even under ordinary conditions upon sensitive organisms exposed to them more or less all the time; for electricity is omnipresent around us and must have an effect upon sensitive natures, though it is generally not observed. This reminds us of the thorough investigations of Reichenbach, some forty years ago, which he preserved in his large work, *Der sensitive Mensch*, and in some more writings before and after its publication. (*Der sensitive Mensch und sein Verhalten zum Ode. Stuttgart. Cotta-scher Verlag*, 1854. 2 vols.)

Already in 1862 Reichenbach showed this emanation of light in complete darkness from a large quartz crystal, directed with its negative end to a photographic plate for fifteen minutes, producing the picture of a cross cut out of a piece of pasteboard which was placed upon the plate. Still more interesting was his experiment to show the light emanating from the fingers of the right hands of five men, placed upon a glass bar about one and one-half inch long, and directed with one end upon a similar cross over a photographic plate for seven and one-half minutes in the dark chamber, by a picture of a brown color of the cross upon the plate. At this time (1862) he offered to the Berlin professors sixteen experiments in all of which he could show light to emanate from the walls and ceilings of the rooms, the points of crystals, the poles of magnets, the organism of man, especially the finger-tips, from chemical action, friction, amorphous masses of metals, triturated kitchen salt, the focus of a lens ; but these noble professors disarranged his arrangements and frustrated his design in the old allœopathic crooked way. (*Odische Begebenheiten zu Berlin in* 1861 *und* 1862. Schroeder. Berlin, 1862.)

Now these emanations of light from these various sources, which can be seen by sensitives in the dark as luminous phenomena, can equally act upon the sensitive photographic plate in the dark and produce pictures upon it as the common daylight does.

It has already been surmised that Rœntgen's discovery will have an important influence upon diagnostics in medicine as the value of it is already acknowledged in surgery. But also here Reichenbach is more than forty years in advance (*Der sens. Mensch,* § 2252) : "Mrs. K. found it amusing to bring the back of her fingers so near to the conductor that her tips absorbed the electricity. Thus the fingers became luminous and transparent, as if before a candle-flame, only much purer, so that she could distinguish in them veins, nerves, tendons, fibres of the ligaments, as beyond conception beautiful and fine, that she thought never to have seen anything more beautiful." The remark of Reichenbach on this occasion was prophetic, and has materialized already at the present day : "This can become an object of incalculable importance for the healing art, especially for diagnostics. It will succeed in making the whole sick organism transparent for high sensitives, and it will be possible to see which internal organs are diseased, and which progress the disease may make, forward or backward. But also the processes in the healthy body will be examined in this manner." The great work of Reichenbach, containing thousands of carefully made experiments, arranged and commented on in the true scientific spirit, and serving as a model of scientific research, was denounced by Dubois Reymond, a late rector magnificus of the great Berlin University, as "one of the most deplorable aberrations to which for a long time a human brain has fallen a victim; fables which deserve to be thrown into the fire." Well, in the whirligig of time the magnificent professor's condemnation came to be executed, but in another sense than he dreamed of in his philosophy. Reichenbach's fables are fired into the Rœntgen rays, and teach the wise men that not all wisdom emanates from the big schools of learning. Nay, since that very discovery of the X-rays, a magnetician, Tormin, has obtained photographic pictures from emanation of light from his finger-tips of the right hand pressed upon the cover of a closed wooden case, in which was contained a photographic plate, within forty-five minutes. (Ludwig Tormin, *Magische Strahlen.* Düsseldorf, 1896.)

You ask, what have these interesting discoveries to do with Homœopathy? Where is here the principle of *Similia Similibus*, the first enunciation of which by Hahnemann a hundred years ago we to-day celebrate? The reason of presenting these few experiments is to show how the elimination of matter enables the forces carried by it as their vehicle to exert their specific energies. As in the light first discovered by Reichenbach, emanating from all matter in light and darkness, which he called Od, and now in the light emanating from the electric cathode in the vacuum and darkness, breaks loose from the crude mass and assumes new properties in the transference from crudity of air to fineness, so also the medicinal forces are liberated from the crude vehicle confining them, and by distributing through and transference upon inert vehicular masses, unfold their specific power when brought in contact with the organism of man in the necessary proportion indicated by the state of the life-force in its pathogenesis through the Hahnemannian law of Similia. Nay, more; the potentiation of the crude medicinal substance is necessary for rendering it homœopathic to the system to which they are to be administered. And here is in point Hahnemann's early observation (*Organon*, first ed., § 7): "There must be a healing principle in the medicines; the understanding forebodes it. But its essence is not perceptible in any wise—only its utterances and actions may be deduced by experience." And *Ib.*, § 254: "The action of the healing anti-disease-potencies which are called medicines upon the living human body happens in such a penetrating manner, it spreads from the point of the fibre endowed with nerves upon which the medicine is first applied with such an incomprehensible rapidity and universality through all the parts of the living individual, that their action might be called almost spiritual as vitality itself, from which its action is reflected upon the organism; the body, animated by irritability and sensation, receiving its specific impression, lends to this action a kind of life."

This spirit-like, dynamic, life-like action of the medicine potentiated on the Hahnemannian plan is certainly as similar to the vitality reflected upon the organism, which he later termed the life-force, as the symptoms of disease to be cured by remedies capable of producing similar symptoms on the healthy. Such fine preparations of medicines as Hahnemann had in his mind's eye were not to be compared to any preparations emanating from the pharmacology of the physico-chemical school. They have nothing in common with them, because no methods known to physical and chemical science are able to detect the least particle of matter in them, not even a molecule, nor less an atom, nor even one calculated by the greatest mathematicians of the age, because they all are left behind in the mode of potentiation, which leads to the conclusion that the crude substance containing the medicinal, "almost spiritual" force, as a mere vehicle, allows it by the method of transference to medicate an enormous amount of inert material, which now, as another vehicle of matter, keeps the imparted medicinal force in a high potency for the use in disease for the sake of healing, and in health for the sake of proving. This is, therefore, the *Similia Similibus*, which, by the new principle enunciated one hundred years ago, was the necessary consequence of its application in practice, and cannot be omitted when celebrating the *Similia Similibus* in regard to the similar symptoms. This unity of Similia, carried into practice, and forming the nucleus for further scientific investigation, forms the eminent problem which the International Hahnemannian Association has to solve. May all the members conceive it deeply in their minds, because it grows out of the true Hahnemannian conception of HOMŒOPATHICS!

*Ceterum censeo, macrodosiam esse delendam.*

